# Whats, hows and whys of programmed DNA elimination in *Tetrahymena*

**DOI:** 10.1098/rsob.170172

**Published:** 2017-10-11

**Authors:** Tomoko Noto, Kazufumi Mochizuki

**Affiliations:** Institute of Human Genetics, UMR 9002, CNRS and University of Montpellier, Montpellier, France

**Keywords:** epigenetics, RNAi, DNA elimination, transposon, *Tetrahymena*

## Abstract

Programmed genome rearrangements in ciliates provide fascinating examples of flexible epigenetic genome regulations and important insights into the interaction between transposable elements (TEs) and host genomes. DNA elimination in *Tetrahymena thermophila* removes approximately 12 000 internal eliminated sequences (IESs), which correspond to one-third of the genome, when the somatic macronucleus (MAC) differentiates from the germline micronucleus (MIC). More than half of the IESs, many of which show high similarity to TEs, are targeted for elimination in *cis* by the small RNA-mediated genome comparison of the MIC to the MAC. Other IESs are targeted for elimination in *trans* by the same small RNAs through repetitive sequences. Furthermore, the small RNA–heterochromatin feedback loop ensures robust DNA elimination. Here, we review an updated picture of the DNA elimination mechanism, discuss the physiological and evolutionary roles of DNA elimination, and outline the key questions that remain unanswered.

## 1. Introduction

Ciliated protozoa undergo extensive programmed genome rearrangements when the germline micronucleus (MIC) produces the new macronucleus (MAC) during sexual reproduction. In this process, many transposon-related sequences are removed from the MAC [1,2]. RNAi-related mechanisms play important roles in programmed genome rearrangements in at least three different ciliates, including *Oxytricha trifallax* [3,4], *Paramecium tetraurelia* [5–7] and *Tetrahymena thermophila* [8–11]. There are substantial (and very interesting) differences in the mechanisms that regulate programmed genome rearrangement in ciliates. This review aims to overview the mechanisms and the roles of programmed genome rearrangement in *Tetrahymena thermophila* (hereafter referred to as *Tetrahymena*)*.* We refer the reader to previous reviews [12–16] for programmed genome rearrangements in other ciliates.

Although it has long been known that programmed genome rearrangements occur genome-wide and eliminate many sequences related to transposable elements (TEs), a global landscape concerning ‘what’ is eliminated—their numbers, chromosomal distributions and relationship with TEs—became available only after the recent establishment of the assemblies of nearly full-length MIC chromosomes [1]. In §2, we overview the programmed genome rearrangements of *Tetrahymena* in the context of the MIC genome.

Current knowledge of the eliminated sequences in combination with the accumulated genetic and biochemical data on the molecular machineries that regulate small RNA-mediated DNA elimination now provide a clear picture of ‘how’ eliminated sequences are recognized. In §3, we discuss how small RNAs target particular sequences for DNA elimination by a complex genome-wide network.

Whenever we present at conferences, the most frequently asked question is, ‘Why do ciliates perform DNA elimination?’ Dr Martin Gorovsky, our former supervisor, taught us not to ask *why* things happen but *how* they occur. We have faithfully followed this advice for over a decade. However, with a recently improved understanding of the DNA elimination pathway, we think that it is time to consider the ‘why’ question because we can now form testable hypotheses (sorry, Marty). In §4, we discuss why *Tetrahymena* perform DNA elimination from the point of view of genome integrity and dynamics.

## 2. The genome of *Tetrahymena*: what is eliminated from the MAC?

### 2.1. The life of *Tetrahymena*

*Tetrahymena* is a free-living freshwater ciliate that is one of the most commonly studied protozoa in laboratories. Like most other ciliates, *Tetrahymena* display nuclear dimorphism [17] through the presence of a diploid germline MIC and a polyploid somatic MAC. Gene expression occurs in the MAC, whereas the MIC is transcriptionally inert (although there is an exception during early sexual reproduction; see below). Because the MAC in G1 phase was reported to contain approximately 23 times more DNA than the G1 MIC [18] and the MIC genome is approximately 1.5 times larger than the MAC genome (see below), the copy number of the MAC chromosomes in G1 phase is estimated to be approximately 70 [19]. However, this number does not agree well with the estimates (approx. 45 C) from the kinetics of phenotypic assortment [20,21], the process in which chromosome copies distribute randomly during MAC vegetative division. Recent sequencing and/or microscopic technologies would aid to reconcile this discrepancy.

**Figure 1.**
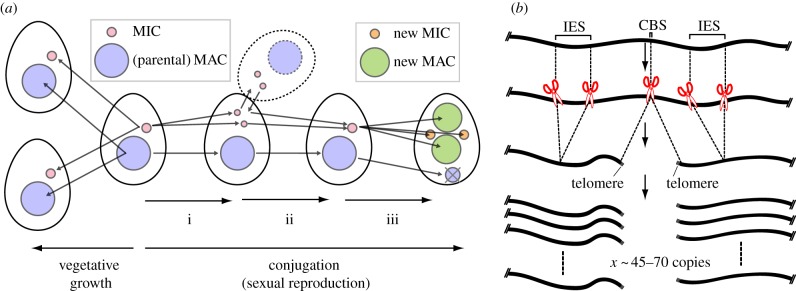
Life cycle and programmed genome rearrangements of *Tetrahymena.* (*a*) Life cycle of *Tetrahymena*. A single *Tetrahymena* cell contains a macronucleus (MAC) and a micronucleus (MIC). When sufficient nutrients are available, *Tetrahymena* grow by binary fission, and the MAC and the MIC divide independently (vegetative growth). After prolonged starvation, cells undergo conjugation, the sexual reproduction process: (i) the MIC undergoes meiosis to generate four haploid nuclei, three of which are discarded, and one selected haploid nucleus proceeds with post-meiotic division to form two haploid pronuclei; (ii) one of the two pronuclei is exchanged between the mating partner, and the stationary and the exchanged pronuclei fuse to form a diploid zygotic nucleus; and (iii) the zygotic nucleus divides twice to form two new MACs and two new MICs, while the parental MAC is degraded. Readers interested in the details of the *Tetrahymena* life cycle should refer to [22]. (*b*) Programmed genome rearrangements. In the newly formed MAC, chromosome breaks occur at CBSs and telomeres are formed *de novo*. In addition, internal eliminated sequences (IESs) are removed by DNA elimination. Each MAC chromosome is endoreplicated to approximately 45–70 copies (see text for discrepancy among the previous estimates of the MAC chromosome copy number).

Figure 1*a* summarizes the life cycle of *Tetrahymena*. During vegetative growth, both the MAC and the MIC divide and segregate to the daughter cells. During conjugation, the sexual reproduction process of *Tetrahymena*, the MIC undergoes meiosis, which is followed by fertilization to form the zygotic nucleus. The zygotic nucleus further divides to make the new MAC and the new MIC. The parental MAC is destroyed by autophagic degradation at the end of sexual reproduction [23]. The new MAC genome is endoreplicated to 4 C immediately after its development, and then programmed genome rearrangements occur concomitant with the second round of endoreplication (4 C to 8 C) [24]. However, endoreplication is not necessary for genome rearrangement [25]. Further endoreplication of the MAC genome occurs later during vegetative growth.

### 2.2. The eliminated sequences

The latest studies on genome sequencing indicate that the sizes of the MIC and MAC genomes are 157 Mb and 103 Mb, respectively [1,26–28]. This difference in size is caused by the two types of programmed genome rearrangements that occur during the development of the MAC.

The first type of programmed genome rearrangement is chromosome breakage at the chromosome breakage sequences (CBSs), accompanied by short DNA trimming and *de novo* telomere formation (figure 1*b*). Because CBSs share a conserved 15 bp element [29], a yet unidentified endonuclease that specifically recognizes CBS probably catalyses the chromosome breakage. The telomere end binding protein homologue Pot2p localizes to CBSs exclusively at the time of chromosome breakage and is suggested to be involved in *de novo* telomere formation [30]. Chromosome breakages split 5 MIC chromosomes into approximately 230 MAC chromosomes, while approximately 50 of them, called the non-maintained chromosomes (NMCs, also called the eliminated mini-chromosomes), are either immediately degraded before telomere addition or lost within approximately 20 fissions during vegetative growth after telomere addition. The mechanism for the disappearance of NMCs is not known. The identified NMCs are generally short, ranging from 30 to 80 kbp, compared with the other MAC chromosomes (40 kb to 1.4 Mb) [1,26,31].

The second type of programmed genome rearrangement is the elimination of internal DNA segments, called internal eliminated sequence (IES), followed by the ligation of flanking MAC-destined sequences (figure 1*b*). Hereafter, we refer to this genome rearrangement event as DNA elimination. In the articles published before 2012, the number of IESs in the *Tetrahymena* MIC was estimated to be approximately 6000. This was based on estimations from a limited sampling of the MIC genome [32] or low coverage sequencing of the MIC genome [33]. With the near-complete MIC genome sequence, the predicted number of IESs has been substantially increased and the latest estimation is approximately 12 000 [1], which corresponds to approximately 46 Mb. The predicted IESs range from 136 bp to 43.4 kb with the mean and the median length of 3.8 and 2.8 kb, respectively.

The excisions of nearly all IESs are believed to be catalysed by the domesticated piggyBac transposase Tpb2p. However, due to technical difficulties, it has not been possible to completely remove Tpb2p activity from the cell [34,35], and thus the absolute requirement of Tpb2p for individual DNA elimination remains elusive. Tpb2p-dependent IESs are imprecisely excised from the genome. End-point heterogeneities of boundaries, mostly within a few base pairs, were observed between siblings [1]. Accordingly, Tpb2p-dependent IESs are found within intergenic regions and introns. In contrast to the majority of IESs, the excision of 12 (and possibly slightly more) IESs is dependent on the other domesticated piggyBac transposases Tpb1p and Tpb6p [36,37]. All known Tpb1/6p-dependent IESs is within exons of protein-encoding genes and precisely excised [1,36,37]. These exceptional 12 IESs share a conserved 12 bp terminal repeat and produce few scnRNAs, if any [36,37]. Therefore, in contrast to the Tpb2p-dependent IESs that are targeted for DNA elimination by an RNAi-related mechanism (see below), these 12 IESs are probably recognized and targeted for DNA elimination directly by Tpb1/6p, and are excluded from IESs in the following discussion.

The majority of the genome that is eliminated from the developing MAC (157 Mb [MIC] − 103 Mb [MAC] = 54 Mb) is occupied by the predicted IESs (46 Mb). We speculate that the most of the remaining 8 Mb also consists of IESs, which were not predicted due to the insufficient quality of the current MIC genome assembly. The contribution of NMCs to genome down-sizing is limited because the sum of identified NMCs totals 0.56 Mb [31], although unidentified large NMCs may exist.

### 2.3. Kinship between transposable elements and IESs

The currently assembled MIC genome contains at least approximately 19 Mb of transposable element (TE)-related sequences [1]. Among the classifiable TE sequences, the majority (91%) correspond to DNA transposons, including Tc1/Mariner, Helitron and Maverick/Tlr families. A small portion (9%) of TEs consists of retrotransposons, all of which are related to non-LTR elements [1]. Approximately 18 Mb of these TE-related sequences are removed from the MAC genome. There is a retention bias toward the terminal regions of TEs, suggesting that occasionally IES elimination incompletely removes TEs, leaving the terminal regions of the TEs in the MAC [1]. Therefore, some (or most) of the approximately 1 Mb TE-related sequences that remain in the MAC are likely to be non-functional fragments of TEs.

Some IESs have intact open-reading frames and terminal repeats of TEs and are found in multiple copies in the MIC genome [1]. Therefore, some IESs are likely to be the result of recent TE activity. Consistently, IES positions are highly variable between closely related *Tetrahymena* species [38]. Many IESs consist of modules of sequences homologous to different TE families, which is probably a consequence of frequent TE insertions into an IES and its following degeneration [1,39]. Some or many regions of IESs that do not show detectable homology with TEs might also be remnants of TEs.

## 3. The molecular mechanism of DNA elimination: how do small RNAs regulate DNA elimination?

It has been known that DNA elimination is regulated by the small RNA-mediated comparison of the MIC to the MAC genomes [8]. Although this view is still valid, recent studies have indicated that additional layers of mechanisms act in addition to the genome comparison. In §3.1, we discuss how a small RNA-based system harnesses nuclear dimorphism and the modular organization of IESs to robustly but flexibly regulate DNA elimination. Next, the molecular players behind this small RNA-mediated regulation are discussed in §3.2. Finally, in §3.3 we outline the unanswered questions regarding the recently discovered small RNA–heterochromatin positive feedback loop in DNA elimination.

### 3.1. Systems point of view of small RNA-directed DNA elimination

**Figure 2.**
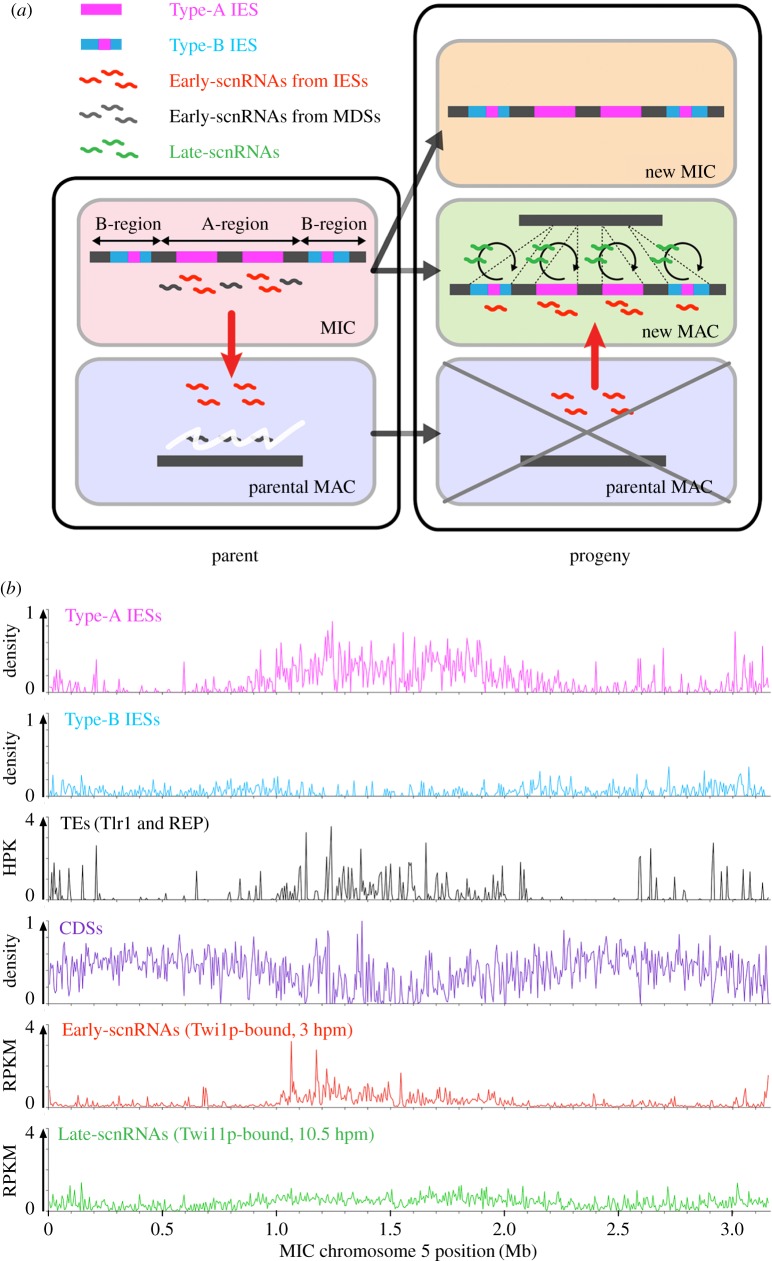
Regulation of DNA elimination by small RNAs. (*a*) A model for small RNA-directed DNA elimination. Early-scnRNAs are expressed from Type-A IESs and their surrounding regions (A-region) in the MIC and then move into the parental MAC, where Early-scnRNAs derived from MAC-destined sequences are degraded (left). When the new MAC is formed in the progeny (right), Early-scnRNAs move from the parental to the new MAC. There, Early-scnRNAs recognize not only Type-A IESs but also Type-B IESs in *trans* through common repetitive sequences. This recognition triggers Late-scnRNA production in *cis*. Early- and Late-scnRNAs cooperatively induce DNA elimination. (*b*) Localizations of Type-A and Type-B IESs, transposons (TEs), and coding sequences and productions of Early- and Late-scnRNAs in the MIC chromosome 5 are shown in histograms with 50 kb bins. Tlr1- and REP-elements were used as TEs. Twi1p-bound 26–32-nt RNAs at 3 hpm and Twi11p-bound 26–32-nt RNAs at 10.5 hpm were analysed as Early- and Late-scnRNAs, respectively.

Here we present a small RNA-centric model of DNA elimination (figure 2*a*) that is based on genetic and biochemical studies of RNAi machineries and high-throughput sequencing of small RNAs [8–11,40–43].

In the early stages of conjugation in the MIC, approximately 3 h post-mixing (hpm), small RNAs of approximately 29 nt, called Early-scnRNAs, are produced from certain (approx. 60%) IESs (called Type-A IESs) and their surrounding regions, whereas the remaining (approx. 40%) IESs (called Type-B IESs) do not produce Early-scnRNAs [43]. Type-A IESs are primarily located at the middle (peri-centromeric) and the end (telomeric) regions of the MIC chromosomes, which are called A-regions, whereas Type-B IESs are primarily located at the chromosomal arms, called B-regions [1] (figure 2*b*). Therefore, contrary to previous ‘scnRNA models’ [8,44], Early-scnRNAs are produced heterogeneously from the MIC genome. There are two biases in the pattern of Early-scnRNA production: the global bias in which Early-scnRNAs are preferentially produced from the A-regions, and the local bias in which, within the A-regions, Early-scnRNAs are preferentially produced from IESs [43].

Early-scnRNAs then localize to the parental MAC at the mid stages of conjugation (approx. 3–7 hpm), where any Early-scnRNAs that are complementary to the MAC genome (i.e. non-IES sequences) are degraded by the process called scnRNA selection [40,42,43], as previously proposed [8,44]. As a result, only Early-scnRNAs that are complementary to the MIC-limited sequences are retained.

Next, the selected Early-scnRNAs translocate to the newly formed MAC (approx. 7 hpm), where they interact with Type-A IESs to induce heterochromatin formation [8,45,46]. Type-B IESs contain repetitive sequences, named A-repeats, which are complementary to Type-A IESs [43]. Thus, Early-scnRNAs also interact with Type-B IESs in *trans* via A-repeats to induce heterochromatin formation. Moreover, Early-scnRNAs induce the production of another group of approximately 29-nt small RNAs, called Late-scnRNAs, in *cis* at both Type-A and Type-B IESs [43]. Late-scnRNAs further trigger heterochromatin formation in *trans*, forming an RNAi-heterochromatin positive feedback loop [43]. The heterochromatinized IESs are eventually excised by Tpb2p [34,35]. When the accumulation of Late-scnRNAs was inhibited by genetically removing zygotically expressed Twi1p and Twi11p (see below), DNA eliminations of Type-A IESs were only weakly inhibited and those of Type-B IESs were more strongly, but not completely, inhibited [43]. These indicate that Early-scnRNAs can directly induce DNA elimination but Late-scnRNAs are necessary to ensure complete removal of all IES copies from the MAC.

The RNAi–heterochromatin positive feedback loop, which is composed of *trans*-recognition and *cis*-spreading mechanisms, probably provides robustness to the system. Our simulations indicated that the feedback loop mechanism can secure the elimination of most IESs, even when 99% of Type-A IESs fails to express Early-scnRNAs [43]. Most of the potentially active TEs are found in Type-A IESs [43]. Active TE-containing (i.e. young) IESs may be constrained by natural selection to localize at the gene-poor A-regions (figure 2*b*), where the production of Early-scnRNAs ensures that their DNA is eliminated from the new MAC. As TE sequences degenerate (i.e. become older), IESs may be tolerated as residents of the gene-rich chromosomal arm regions and then transition into Type-B IESs.

### 3.2. Mechanistic point of view of small RNA-directed DNA elimination

**Figure 3.**
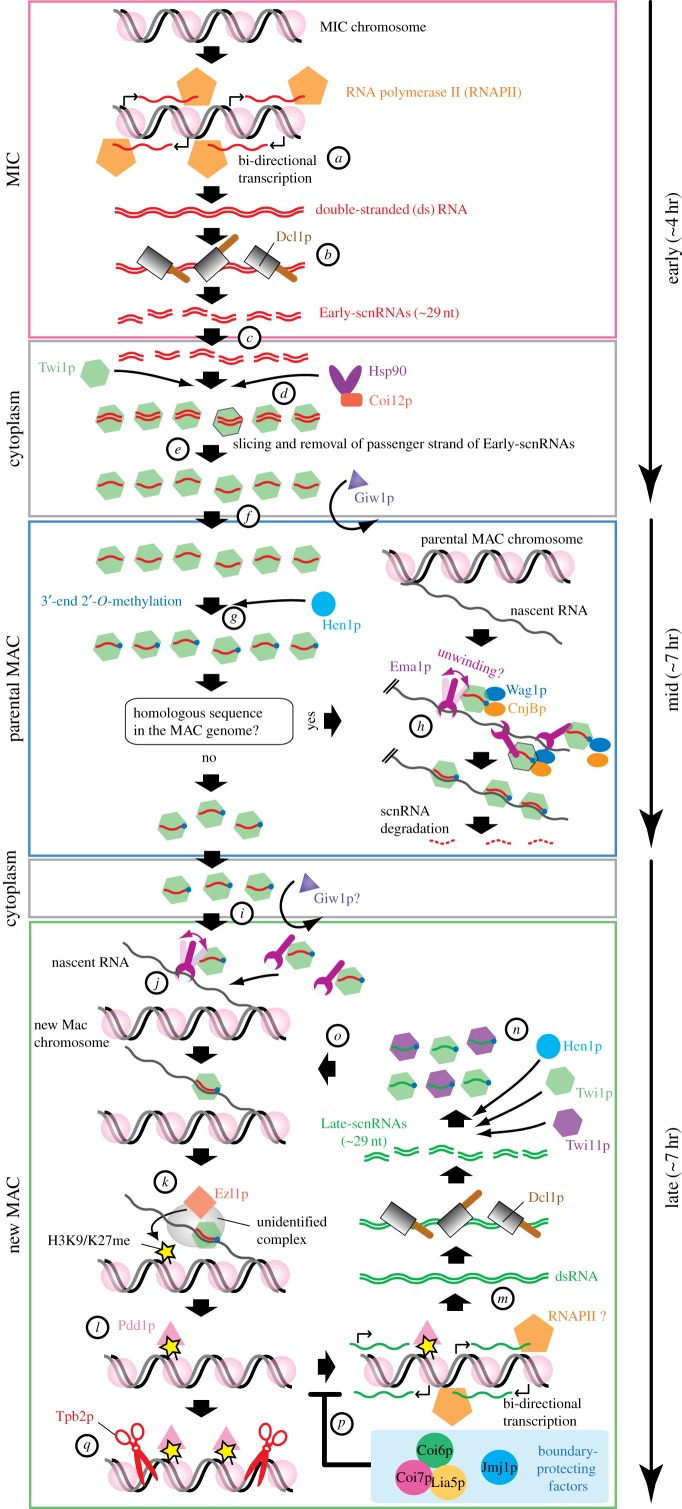
Machinery of DNA elimination. Bi-directional transcription in the MIC by RNA polymerase II produces double-stranded RNAs (*a*), which are processed by the Dicer-like protein Dcl1p to Early-scnRNAs (*b*). Early-scnRNAs are exported to the cytoplasm (*c*) where they are loaded into the Argonaute protein Twi1p with the aid of Hsp90 and its co-chaperone Coi12p (*d*). The passenger strand of the Early-scnRNA duplex is cut and removed by the Slicer activity of Twi1p (*e*). Giw1p transports the Early-scnRNA–Twi1p complex to the parental MAC (*f*). The RNA methyltransferase Hen1p introduces 2′-*O*-methylation to the last nucleotides of Early-scnRNAs (*g*). The RNA helicase Ema1p facilitates the interaction between the Early-scnRNA–Twi1p complex and complementary nascent transcripts, which induce the degradation of Early-scnRNAs (scnRNA selection). Two GW-repeat proteins, Wag1p and CnjBp, are also necessary for scnRNA selection (*h*). The remaining Early-scnRNA–Twi1p complexes are transported into the new MAC, possibly by Giw1p (*i*). Like in the parental MAC, Ema1p facilitates the interaction between the Early-scnRNA–Twi1p complex and nascent RNAs (*j*). This interaction recruits the histone methyltransferase Ezl1p, which catalyses H3K9me3 and H3K27me3 (*k*). The HP1-like protein Pdd1p binds to these methylated histones (*l*). Heterochromatin induces bi-directional transcription in *cis*, resulting in Dcl1p-dependent production of Late-scnRNAs (*m*). Late-scnRNAs are loaded into Twi1p and Twi11p and then are 2′-*O*-methylated by Hen1p (*n*). The Late-scnRNA-Twi1/11p complex recruits Ezl1p, forming an RNAi-heterochromatin positive feedback loop (*o*). The feedback loop is downregulated by ‘boundary-protecting factors’ Coi6p (HP1-like), Coi7p, Lia5p (domesticated piggyBac transposase) and Jmj1p (H3K27 demethylase) (*p*). The domesticated piggyBac transposase Tpb2p eventually excises heterochromatinized IESs (*q*).

The molecular machineries of small RNA-directed DNA elimination are illustrated in figure 3. The MIC is transcriptionally inert during all life stages, except in meiotic prophase [47,48], when the MIC genome is transcribed bi-directionally (figure 3*a*) [40,49]. The resulting double-stranded RNAs are processed to Early-scnRNAs by the Dicer protein Dcl1p in the MIC (figure 3*b*) [9,10]. The global and local biases in the pattern of Early-scnRNA production described above are determined at the level of transcription [42]. Because Rpb3p, a RNA polymerase II subunit, localizes to the MIC during meiotic prophase, while Rpc5p, a shared subunit of RNA polymerase I and III, does not [50], the MIC is probably transcribed by RNA polymerase II.

Early-scnRNAs are then exported by a yet unidentified mechanism to the cytoplasm (figure 3*c*) and loaded into the Argonaute protein Twi1p (figure 3*d*) [11,41]. This loading process requires Hsp90 and its co-chaperone [51]. Twi1p has endonuclease (Slicer) activity that cuts and removes one of the two strands of the loaded Early-scnRNAs (figure 3*e*) [41]. Then, the single-stranded Early-scnRNA–Twi1p complex interacts with Giw1p and is imported into the parental MAC (figure 3*f*) [41]. The MAC and the MIC have distinct nuclear pore proteins and use different importins/exportins [52–54]. Exactly how Giw1p links the Twi1p–Early-scnRNA complex to the MAC-specific nuclear import pathway is unclear.

In the parental MAC, Early-scnRNAs that are complementary to the MAC genome are degraded by scnRNA selection (figure 3*h*) [11,40,42]. Although Early-scnRNAs are stabilized by Hen1p-catalysed 2′-*O*-methylation at their 3′-ends (figure 3*g*) [55], this modification does not appear to be involved in scnRNA selection because Early-scnRNAs complementary to the MAC genome were degraded faster than those to IESs even in the absence of Hen1p (T.N. & K.M. 2017, unpublished data). The putative RNA helicase Ema1p triggers Early-scnRNA degradation, probably by promoting the interaction between Early-scnRNA and chromatin through nascent transcripts [40]. The requirement of complementary transcripts for scnRNA selection was directly demonstrated in *Paramecium* by RNAi-knockdown [7]. CnjBp and Wag1p also have a redundant role in scnRNA selection [56]. They both contain GW repeats, also called the AGO hook, which are characteristics of several Argonaute-interacting proteins [57]. Although scnRNA selection is one of the key processes in the regulation of DNA elimination, its molecular mechanism remains elusive. Ema1p, CnjBp and/or Wag1p possibly link Early-scnRNAs to RNA degradation machinery. Alternatively, there may not be specific RNA degradation machinery for scnRNA selection, but it is possible that the Twi1p-binding proteins promote dissociation of Early-scnRNAs from Twi1p, which would allow nonspecific RNase(s) access to Early-scnRNAs.

In parallel to scnRNA selection, the MIC undergoes meiosis, zygote formation, and formation of the new MAC and MIC. When the new MAC develops, those Twi1p–Early-scnRNA complexes that are not degraded by scnRNA selection move from the parental MAC to the new MAC (figure 3*i*) [8,41]. In the new MAC, Early-scnRNA–Twi1p complexes interact with chromatin through nascent transcripts in a Ema1p-dependent manner (figure 3*j*), and, by a yet unknown mechanism, recruit the histone methyltransferase Ezl1p (figure 3*k*). As a result, methylated histones H3 at lysine 9 and lysine 27 (H3K9/K27me3) as well as the HP1-like protein Pdd1p, which binds to these methylated histones, accumulate on Type-A IESs and A-repeats on Type-B IESs (figure 3*l*) [40,43,45,46].

Next, Late-scnRNAs are produced from IESs in the new MAC in an Ezl1p- and Pdd1p-dependent manner (figure 3*m*) and are loaded into the two Argonaute proteins Twi1p and Twi11p (figure 3*n*) [43]. Late-scnRNA and heterochromatin form a positive feedback loop to facilitate Late-scnRNA biogenesis and heterochromatin formation on IESs (figure 3*o*). Because heterochromatin eventually induces DNA excision [34], this activity must be precisely stopped at the borders of the IESs (figure 3*p*). Several items remain unclear, including how heterochromatin components induce Late-scnRNA production, how the borders of IESs are determined, and the functional distinction, if any, between the two Argonaute proteins. These issues are discussed in §3.3.

Heterochromatinized IESs are assembled into large nuclear foci called heterochromatin bodies (HBs) before or during DNA elimination. Pdd1p is hyper-phosphorylated upon heterochromatin establishment [58]. The phosphorylation and subsequent dephosphorylation of Pdd1p are required for the assembly of HBs [59,60]. The dephosphorylation of Pdd1p also promotes RNA–Pdd1p interaction by reducing the net negative charge of Pdd1p [59]. RNAs may act as glue to assemble multiple heterochromatinized IESs into an HB. The RNA-binding aggregation-prone protein Jub6p is also required for HB formation, suggesting that RNA-mediated phase separation may play a role in heterochromatin aggregation [61].

The final excision step of IESs require Tpb2p (except for the 12 Tpb1/6p-dependent IESs) and non-homologous end-joining machinery for their repair steps [34,35,62]. Although Tpb2p can directly interact with H3K9me3, such interaction does not fully explain how Tpb2p executes DNA excision at the IES border, but not within the body of the IES. Tpb2p may become active only when it interacts with a specific chromatin environment at the IES border.

### 3.3. Three unknowns of the small RNA–heterochromatin positive feedback loop

#### 3.3.1. What mechanism drives the small RNA–heterochromatin positive feedback loop?

How does heterochromatin induce Late-scnRNA production? Clearly, the production of Late-scnRNAs is preceded by the transcription of their precursors. A heterochromatin component may directly promote bi-directional transcription. While transcriptional enhancement by heterochromatin may not be intuitive, heterochromatin-induced transcription has been observed in different eukaryotes [63,64]. Alternatively, promiscuous transcription may occur independently of heterochromatin and a heterochromatin component may direct the transcripts to the Late-scnRNA-producing pathway.

We hypothesize that there are at least two consecutive heterochromatin states on an IES: heterochromatin state I, which induces the production of Late-scnRNAs; and heterochromatin state II, which induces heterochromatin aggregation and subsequent DNA elimination. Over 20 heterochromatin components have been reported to be involved in DNA elimination [19,59]. Investigations of chromatin localization with high temporal resolution would provide a basis to understand how different heterochromatin states exert distinct biological effects.

The phosphorylation states of Pdd1p are correlated with the two heterochromatin states: Pdd1p is hyper-phosphorylated when Late-scnRNAs are produced and hypo-phosphorylated when DNA elimination begins [58,59]. Although the phosphorylation of Pdd1p is probably not necessary for the biogenesis of Late-scnRNAs [60], enzymes regulating Pdd1p phosphorylation might also regulate Late-scnRNA production through another heterochromatin component. Future studies should identify kinase(s) and phosphatase(s) of Pdd1p to determine if they play a role in Late-scnRNA production.

#### 3.3.2. How is the positive feedback loop downregulated at the ends of IESs?

**Figure 4.**
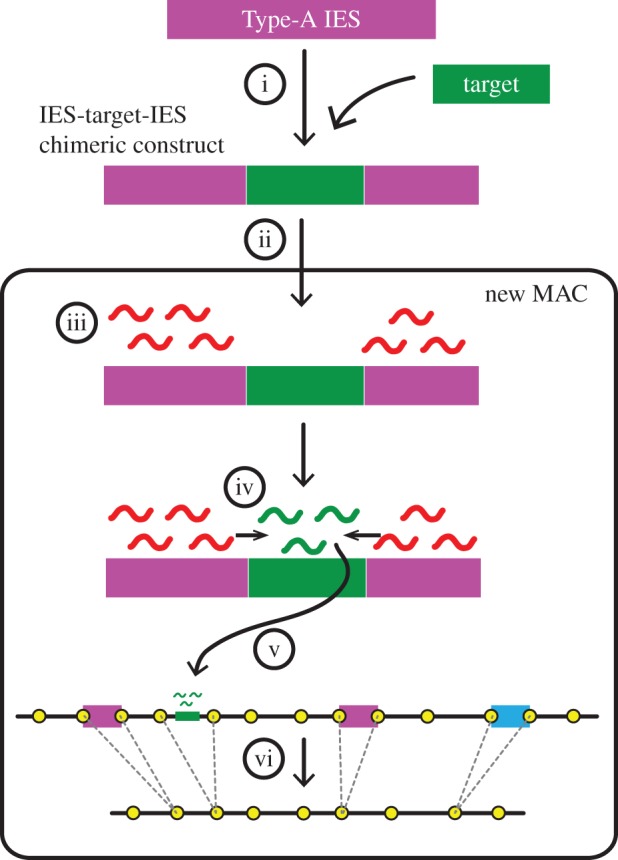
Co-deletion (coDel). A DNA construct in which a non-IES sequence (target) is inserted into the middle of a Type-A IES (i) is introduced into the developing MAC (ii). The Type-A IES of the construct is recognized by Early-scnRNAs that are produced from the corresponding endogenous Type-A IES (iii) and trigger Late-scnRNA production from the adjacent target sequence in *cis* (iv). Late-scnRNAs then recognize the endogenous target locus in *trans* (v) and induce ectopic DNA elimination (vi). Yellow circles indicate the hypothetical IRHs.

Exactly how the borders of IESs are determined is still unclear because no consensus sequence has been identified around the IES boundaries. An implication can be obtained from the phenomenon of co-deletion (coDel), which involves targeted ectopic DNA elimination that is induced at any non-IES sequence by introducing an IES–target–IES chimeric construct into the new MAC [43,65]. The *cis*-spreading of the RNAi–heterochromatin signal and *trans*-recognition by scnRNAs are the bases of coDel (figure 4). Although the boundaries of coDel-induced elimination sites are not as homogeneous as those of IESs, there appear to be preferred locations for their boundaries [65]. We hypothesize that there are insulators of RNAi–heterochromatin *cis*-spreading (IRHs) at the IES boundaries as well as at non-IES loci in the MIC genome (figure 4, yellow circles). When a non-IES region is targeted by coDel, two IRHs adjacent to the target may insulate the spreading of the RNAi–heterochromatin feedback loop, and thus DNA excision occurs at the two IRHs. Identification of potential IRHSs by a large-scale coDel screening may allow us to extract sequence features of IRHs, which may provide insight into this long-standing mystery.

It has been reported that some IESs have *cis*-acting elements that play an important role in determining the borders of IESs [66,67]. A subset of *cis*-acting elements potentially forms a G-quadruplex structure. Additionally, the G-quadruplex binding protein Lia3p is important for properly determining the boundaries of G-quadruplex-associated IESs [66]. It is important to investigate the role of Lia3p, the G-quadruplex, and the other known *cis*-acting elements in limiting the proliferation of Late-scnRNA production and/or heterochromatin formation in the new MAC. Additionally, the relationship between the *cis*-acting elements and the hypothetical IRHs should be analysed.

The HP1-like protein Coi6p (aka Hpl1p [68]), the H3K27 demethylase Jmj1p, and the two Coi6p-binding proteins Coi7p and Lia5p are important in the process of confining Late-scnRNA production and heterochromatin to IESs [69,70]. Coi6p and Jmj1p may counteract the interaction of Pdd1p and H3K9/K27me3 by competing and removing H3K27me3, and thus negatively regulate heterochromatin proliferation. Coi7p is necessary for the stable accumulation of Coi6p. Lia5p is an enzymatically inactive piggyBac transposase [71] and may directly interact to DNA to perform the insulating function. It will be important to determine the chromosomal localizations of these ‘boundary-protecting factors’ with high spatio-temporal resolution.

#### 3.3.3. What are the roles of the two late-scnRNA-loaded Argonaute proteins?

The two Argonaute proteins Twi1p and Twi11p are involved in DNA elimination. Twi1p is expressed both maternally from the parental MAC and zygotically from the new MAC. In contrast, Twi11p is exclusively zygotically expressed from the new MAC. As a consequence, the maternal Twi1p interacts with Early-scnRNAs while the zygotic Twi1p and Twi11p are loaded with Late-scnRNAs [43]. The maternal Twi1p and Early-scnRNAs are sufficient to induce DNA elimination of the majority of IES copies, whereas the zygotic Twi1p/Twi11p and Late-scnRNAs are important for completing DNA elimination. Loss of both zygotic Twi1p and Twi11p cause mild DNA elimination defects in Type-A IESs and severe elimination defects in Type-B IESs [8,11,43].

Currently, it is unclear if the zygotic Twi1p and Twi11p have distinct roles. Although Twi1p possesses Slicer activity, Twi11p lacks the Slicer conserved catalytic core. The Slicer activity of Twi1p potentially enhances the release of Twi1p from the chromatin, and thus may have a negative effect on heterochromatin formation. On the other hand, Twi11p may remain longer at chromatin and more efficiently promote heterochromatin formation. Therefore, Twi1p and Twi11p might have opposing roles in the small RNA–heterochromatin positive feedback loop. The difference in the timing of their zygotic expression (*TWI11* appears first and then zygotic *TWI1* accumulates [43]) might reflect this functional difference. Future genetic and biochemical analyses may reveal a functional divergence between Twi1p and Twi11p.

## 4. The evolutionary advantage of DNA elimination: why do *Tetrahymena* perform DNA elimination?

In this section, we discuss a fundamental question: why do *Tetrahymena* perform DNA elimination? Because a large fraction of IESs are related to TEs, and RNAi-related mechanisms repress TEs in many eukaryotes, DNA elimination has probably evolved from an ancestral mechanism of transposon silencing. In this sense, *Tetrahymena* perform DNA elimination to defend against TEs. However, the relationship between DNA elimination and TE silencing does not explain why *Tetrahymena* perform DNA elimination because (as in most eukaryotes) TEs can be silenced (instead of eliminated) in the MAC by the RNAi–heterochromatin system.

### 4.1. Why does the MIC maintain IESs?

To address why *Tetrahymena* perform DNA elimination, it is useful to consider the question, ‘Why does the MIC maintain IESs?’ One of the most important functions of the MIC is to form competitive sexual progeny. For this purpose, the MIC should provide genetic variation to enhance the progeny's chance of survival during periods of environmental turmoil. Genome reorganization by TE jumping and recombination at repetitive TEs result in genetic variabilities [72,73]. Additionally, TEs act as genetic reservoirs of new proteins, non-coding RNAs and gene-regulatory sequences, which can be beneficial to the host [74–76]. The MIC may maintain IESs to accelerate genetic diversification.

On the other hand, the MIC must maintain genome integrity and faithfully transmit genetic information to the next generation. The MIC divides via typical mitosis whereas the MAC divides via amitosis. This difference is reflected by the fact that chromosomes of the MIC, but not the MAC, contain centromeres that are associated with the centromeric H3 variant CenH3 (Cna1p) [77,78]. The predicted centromere is located roughly at the middle of each MIC chromosome [1]. CenH3 proteins of *Tetrahymena* and *Paramecium* disappear from the MAC concomitant with DNA elimination, suggesting that sequences associated to the centromere function are removed with some IESs [77–79]. It is also possible that some IESs provide binding sites for other chromatin regulators such as condensins and cohesins [80,81]. Future investigation of the localization of the chromatin regulators in the MIC chromosomes should determine whether and how IESs regulate chromosome segregation.

**Figure 5.**
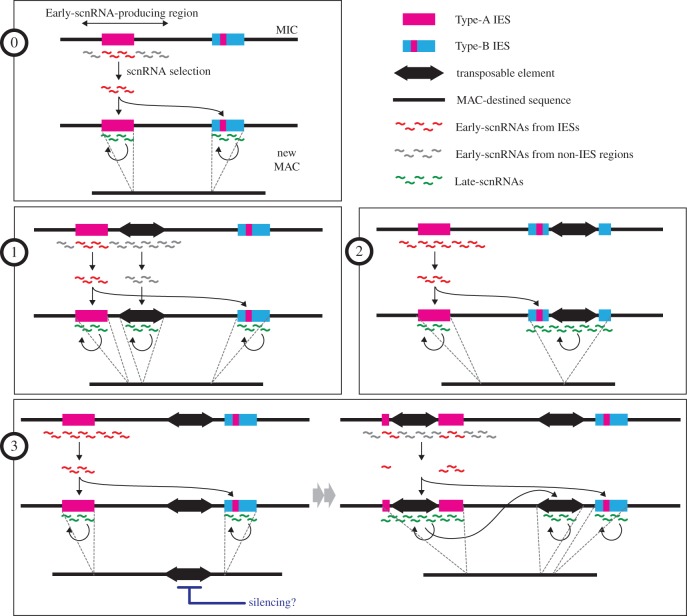
TE invasion and outcomes. Scenario 0: a schematic representation of DNA elimination without naive TE invasion. Scenario 1: a new insertion of a naive TE into an Early-scnRNA-producing region. This results in Early-scnRNA production from the TE and elimination of the TE from the new MAC. Scenario 2: a new insertion of a naive TE into a Type-B IES. This results in production of Late-scnRNAs from the TE by *cis*-spreading and thus the elimination of the TE with the adjacent IES from the new MAC in the next generation. Scenario 3: a new insertion of a naive TE into a non-IES and non-Early-scnRNA-producing region of the MIC genome. This does not immediately provoke the small RNA pathway and the TE may freely jump. The TE also retains in the new MAC (there may be a TE-silencing mechanism in vegetative cells). However, once a copy of the TE is trapped in an IES or Early-scnRNA-producing region (such probability is high as IESs occupy one-third of the MIC genome), Scenario 1 or 2 occurs. Additionally, once this copy produces Early- and/or Late-scnRNAs, all other copies of the TE are recognized in *trans* and targeted for elimination.

The involvement of IESs in proper chromosome segregation explains the roles of only a small fraction of the approximately 12 000 IESs in the 5 MIC chromosomes. Another potential role of IESs in maintaining genome integrity is to suppress invading TEs. By the small RNA-mediated *trans*-recognition explained above, any TE in the MIC genome is subjected to DNA elimination as soon as a copy of the TE jumps into an IES or an Early-scnRNA-producing region (see figure 5 for possible scenarios of naive TE invasion). Perhaps a large portion of the MIC genome is occupied by IESs to efficiently trap invading TEs. It is known that exogenous sequences introduced into non-IES regions of the MIC chromosomes are subjected to elimination in a position-dependent manner [82,83]. This effect is possibly related to the trap effect of Early-scnRNA-producing regions. A systematic study of random transgene insertion into the MIC should aid in the understanding of the positional effects and testing roles of the IESs as TE-traps.

### 4.2. Why do *Tetrahymena* perform DNA elimination?

Let us return to the original question: why does the MAC perform DNA elimination? Because polyploid MAC chromosomes are adapted for amitotic chromosome segregation, they do not need mitotic chromosome segregation. Additionally, as a soma, the MAC does not accumulate heritable genetic changes. Perhaps the MAC performs DNA elimination because nuclear dimorphism and polyploidy allows it to do so, and reduces the cost of DNA replication and continuous surveillance of TEs. Although the effect of DNA elimination on replication is limited in *Tetrahymena* (which eliminates only 30% of the genome from the MAC), elimination is more prevalent in other ciliates, some of which remove over 90% of their genome [84]. DNA elimination may have evolved from an ancestral ciliate that encountered a heavy TE load. Another interesting question is: why do human somatic cells not undergo DNA elimination when approximately 45% of their genome consists of TEs? We speculate that TEs are required for faithful chromosome segregation and genome integrity in diploid somatic cells to avoid aneuploidy and cancer in the context of multicellularity.

**Figure 6.**
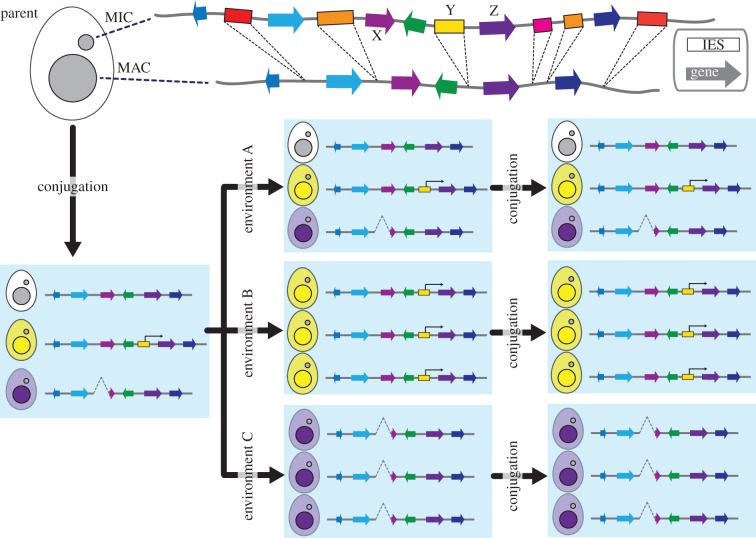
Potential impact of variations in DNA elimination on environmental adaptation. (Top) Eliminations of IESs (boxes) make the MAC genome streamlined. (Left) Spontaneous retention of IES-Y, which promotes the expression of Gene-Z or an ectopic DNA elimination at Gene-X make phenotypic variations. (Middle) These variations have no selective advantage in Environment A. The expression of Gene-Z (yellow cells) provides adaptive advantage in Environment B. The absence of Gene-X (purple cells) is beneficial in Environment C. (Right) The altered pattern of DNA elimination can be *trans*-generationally transmitted through conjugation by scnRNA selection.

DNA elimination also acts as a device for transgenerational epigenetic inheritance, as previously discussed in other ciliates [13,85]. The approximately 12 000 IESs are mostly located in intergenic regions of the gene-dense genome (approx. 27 000 predicted genes, one gene/3.7 kb of the MAC genome) [26]. Therefore, spontaneous DNA elimination ‘errors’ (IES retention or ectopic DNA elimination) could influence the expression of nearby genes in the MAC. Because DNA elimination occurs during the second round of the MAC genome endoreplication [24], DNA elimination potentially forms eight variants per locus. The phenotypic assortment [20,21], in which amitotic chromosome segregation of the polyploid MAC randomly assort a variant, may provide increased fitness to cells that are adapting to environmental change (figure 6). Moreover, the scnRNA selection mechanism allows an advantageous DNA elimination variant to be epigenetically inherited by sexual progeny without phenotypic assortment (figure 6). Future studies should be designed to investigate the frequency of DNA elimination errors, and test if such errors and the following transgenerational epigenetic inheritance indeed aid in environmental adaptation.

## 5. Concluding remarks

TEs are considered necessary evils in genome dynamics and evolution; although they must be tightly downregulated, their activities are the drivers of genome reorganization. As discussed in this review, this balance has probably shaped small RNA-directed DNA elimination in *Tetrahymena*. Although the overall pathway of DNA elimination of *Tetrahymena* has emerged, key questions regarding its molecular mechanism, physiological function and evolutionary significance remain. For example, how are IES-specific Early-scnRNAs selected? How does heterochromatin induce Late-scnRNA production? How are the borders of IESs determined? What are the roles of IESs in the MIC? To what extent do spontaneous DNA elimination errors contribute to environmental adaptation? Further genetic, biochemical and comparative genomic analyses of the DNA elimination mechanism, in the context of TE regulation and genome evolution, should provide an exciting new entry point to answer these long-standing questions.

## Data accessibility

This article has no additional data.
